# Supplementary material for: Yeast Oral Delivery of DAF16 shRNAs Results in Effective Gene Silencing in C. elegans
Source: Curr Issues Mol Biol. 2025 Jul 20;47(7):570. doi: 10.3390/cimb47070570 (PMC12293383; doi:10.3390/cimb47070570)
Supplement: Supplementary file 1 [file cimb-47-00570-s001.zip › cimb-3650010-supplementary.pdf]

**Yeast oral delivery of DAF16 shRNAs results in effective gene silencing in *C. elegans***
Supplemental material

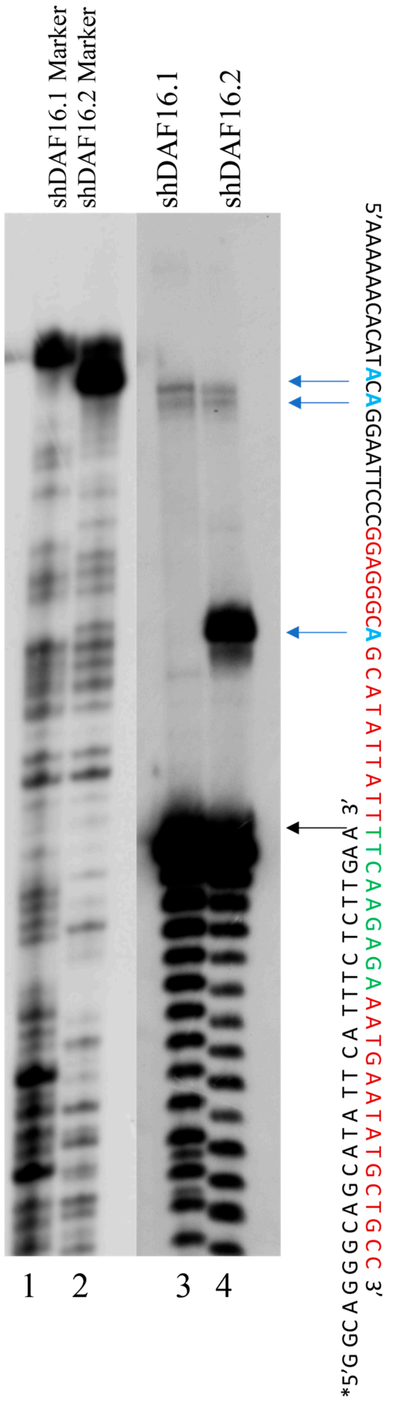


**Figure S1**: Primer extension analysis to determine the transcription start sites (TSSs) of shDAF16.1 and shDAF16.2 (lanes 3 and 4). Blue arrows indicate the putative TSSs. The target and passenger sequence is highlighted in red, the loop sequence in green. The sequence of the oligonucleotides terminally labeled with [$^{32}$P] is also shown. Lanes 1 and 3: size markers corresponding to 60 nucleotides.

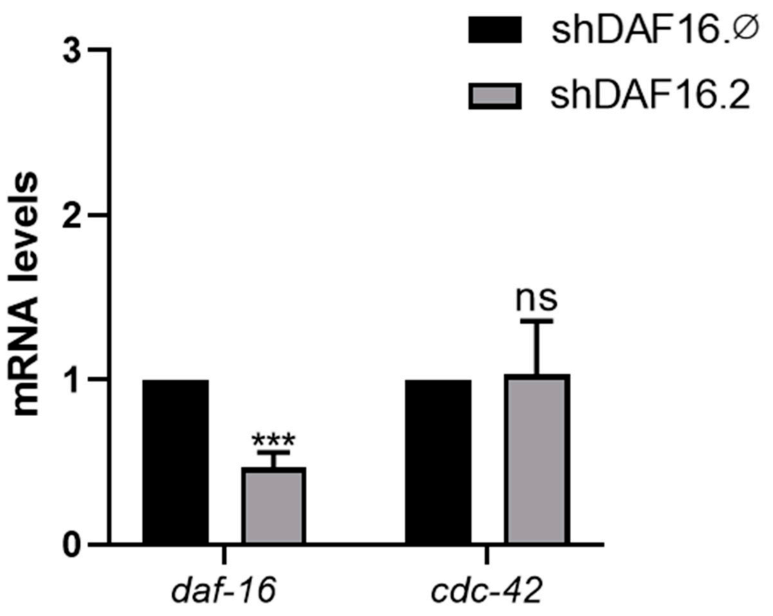


**Figure S2**: RT-qPCR analysis of *daf-16* and *cdc-42* transcript levels in 1-day adult wild type worms treated with the different shRNAs from embryo hatching. Statistical analysis was evaluated by one-way ANOVA with the Bonferroni post-test; asterisks indicate significant differences (***p < 0.001).
